# Preschoolers Benefit Equally From Video Chat, Pseudo-Contingent Video, and Live Book Reading: Implications for Storytime During the Coronavirus Pandemic and Beyond

**DOI:** 10.3389/fpsyg.2020.02158

**Published:** 2020-09-03

**Authors:** Caroline Gaudreau, Yemimah A. King, Rebecca A. Dore, Hannah Puttre, Deborah Nichols, Kathy Hirsh-Pasek, Roberta Michnick Golinkoff

**Affiliations:** ^1^College of Education and Human Development, University of Delaware, Newark, DE, United States; ^2^Human Development and Family Studies, Purdue University, West Lafayette, IN, United States; ^3^College of Education and Human Ecology, The Ohio State University, Columbus, OH, United States; ^4^Wheelock College of Education and Human Development, Boston University, Boston, MA, United States; ^5^Department of Psychology, Temple University, Philadelphia, PA, United States; ^6^The Brookings Institution, Washington, DC, United States

**Keywords:** reading, video chat, vocabulary, online learning, contingency, coronavirus disease, literacy

## Abstract

During the unprecedented coronavirus disease (COVID-19) crisis, virtual education activities have become more prevalent than ever. One activity that many families have incorporated into their routines while at home is virtual storytime, with teachers, grandparents, and other remote adults reading books to children over video chat. The current study asks how dialogic reading over video chat compares to more traditional forms of book reading in promoting story comprehension and vocabulary learning. Fifty-eight 4-year-olds (*M*_age_ = 52.7, *SD* = 4.04, 31 girls) were randomly assigned to one of three conditions (Video chat, Live, and Prerecorded). Across conditions, children were read the same narrative storybook by a female experimenter who used the same 10 scripted dialogic reading prompts during book reading. In the *Video chat* (*n* = 21) and *Live conditions* (*n* = 18), the experimenter gave the scripted prompts and interacted naturally and *contingently*, responding in a timely, relevant manner to children’s behaviors. In the *Prerecorded condition* (*n* = 19), children viewed a video of an experimenter reading the book. The Prerecorded condition was *pseudo-contingent*; the reader posed questions and paused for a set period of time as if to wait for a child’s response. After reading, children completed measures of vocabulary and comprehension. Results revealed no differences between conditions across six different outcome measures, suggesting that children comprehended and learned from the story similarly across book formats. Further, children in the three experimental conditions scored significantly higher on measures than children in a fourth condition (control) who had never read the book, confirming that children learned from the three different book formats. However, children were more responsive to the prompts in the *Live* and *Video chat* conditions than the *Prerecorded* condition, suggesting that children recognized that these interactions were contingent with their responses, a feature that was lacking in the *Prerecorded* condition. Results indicate that children can comprehend books over video chat, suggesting that this technology is a viable option for reading to children, especially during the current pandemic.

## Introduction

So please, oh *please*, we beg, we pray,

Go throw your TV set away,

And in its place you can install

A lovely bookshelf on the wall. – Roald Dahl (1964)

Roald Dahl’s quote from his beloved book, *Charlie and the Chocolate Factory*, illustrates a belief that is still held today by many parents and educators: reading is beneficial for children’s academic success, while time spent watching TV should be limited. The quote is even more relevant today during the current coronavirus disease (COVID-19) pandemic, as caregivers debate how much screen time is allowable for young children while staying at home (e.g., Cheng and Wilkinson, 2020). News reports suggest that many families are engaging in video chatting to keep children connected with family members, teachers, and classmates (Smith, 2020). Some families are also using video chat to engage in *shared book reading* (e.g., Guynn, 2020), a practice where an adult reads a book to a child or group of children and has conversations about the story and related topics (What Works Clearinghouse, 2015). Shared book reading has been linked to a variety of positive outcomes for children, such as increased vocabulary knowledge (Montag et al., 2015), better comprehension of new stories (Clarke et al., 2010), and improved print knowledge (Piasta, et al., 2012). As parents try to navigate the complex world of online educational activities for children during the current stay-at-home orders, research is needed to assess whether virtual shared book reading elicits the same benefits as traditional shared book reading. The current study explored whether preschoolers can learn vocabulary and comprehend stories read to them over video chat.

A large body of research suggests that children benefit most from shared book reading when *dialogic reading* practices are incorporated into reading sessions (e.g., Hargave and Sénéchal, 2000; Strouse et al., 2013). *Dialogic reading* occurs when readers go beyond the text, adding prompts, asking questions, making connections between the book and children’s lives, providing the child with praise, and correcting misunderstandings (Whitehurst et al., 1988; Arnold and Whitehurst, 1994; Zevenbergen and Whitehurst, 2003). Dialogic reading aligns with research-based principles for optimal learning (Hirsh-Pasek et al., 2015b). According to learning scientists, children learn best when they are *active* and *engaged*, in *meaningful contexts*, and are *socially interactive* (Hirsh-Pasek et al., 2015b). When using dialogic reading practices, children are *actively* answering questions and responding to prompts posed by adults. Adults *engage* children by following children’s interests and focusing children’s attention on key points in the story (Hassinger-Das et al., 2019). Adults can also create *meaningful contexts* for children, using “distancing prompts” to relate the story to children’s lives (Hassinger-Das et al., 2017). Lastly, adults and children *interact socially* when engaging in dialogic reading as the reading partners converse about the story and adults aid children in processing the story with additional prompts and explanations.

Dialogic reading practices have been linked to a variety of positive outcomes for children’s reading comprehension and vocabulary learning. For example, Whitehurst et al. (1988) found that when parents were trained to ask their children questions and expand on the story during a 1-month home book reading intervention, children demonstrated higher expressive vocabulary abilities than children in a control. Hargave and Sénéchal (2000) similarly reported greater vocabulary learning in children when parents used dialogic reading techniques than when parents simply read the text. Additionally, when parents use “distancing prompts,” or questions or prompts that relate the story to children’s lives, children comprehend more from book reading (Hassinger-Das et al., 2016).

Children’s *responses* to prompts and questions posed during dialogic reading relate to their learning, as well. For example, Dickinson and Smith (1994) found that using dialogic reading styles in preschool classrooms led to an increase in children’s talk, which in turn predicted an increase in their vocabulary gains. However, although research describes the types of questions and responses shared between adults and children during book reading (e.g., Deshmukh et al., 2019) and children’s accuracy in responding to prompts in different instructional conditions (Walsh and Rose, 2013), there is a surprising lack of research on the relationship between children’s responses during book reading and their learning, as noted by Walsh and Hodge (2016).

The back-and-forth personalized social interactions that are at the core of dialogic reading are also central to how children learn language in general. Indeed, children learn best in one-on-one contexts, in which a caring adult responds to the child and the dyad takes turns responding in back-and-forth communication (Hirsh-Pasek et al., 2015a). This type of communication is characterized as *contingent* – a speaker’s utterance is temporally or topically related to the other speaker’s utterances (Troseth et al., 2006). Research shows that this back-and-forth conversation between adults and children is related to later language ability in children (Hirsh-Pasek et al., 2015a). There is also evidence that back-and-forth interactions between parents and children are related to stronger connectivity in the white matter connecting two central language brain areas (Romeo et al., 2018). Although there are likely multiple mechanisms for these effects, in the domain of book reading, one contributing factor appears to be that the one-on-one context allows an adult to tailor reading to a particular child’s level of understanding, allowing children to learn at their own pace (Connor and Morrison, 2016).

While dialogic reading is a relatively simple practice (Arnold et al., 1994; Blom-Hoffman et al., 2008), there is wide variability in the extent to which parents use these practices when reading with children (Hindman et al., 2014; Troseth et al., 2019) and not all environments and family situations allow for the one-on-one interaction that is at the core of dialogic reading. Parents who are traveling or live apart from children may not be present for book reading on a daily or even weekly basis. The preschool environment is another potential source of rich dialogic reading interaction, but teachers have limited time to read to children individually and build on children’s interests and queries and, in general, teachers rarely engage in extended conversations with individual children (Justice et al., 2008). Dialogic reading may also occur with extended family or other caregivers, but during stay-at-home orders, many children cannot spend time engaging in storybook reading with adults who reside outside of their household, such as babysitters and grandparents. Even within the home, parents may dedicate less time to shared book reading during a pandemic. Working from home with much of their time balancing work and caring for children leaves many parents with little time for rich bouts of dialogic reading. In families where parents are deemed essential workers (e.g., healthcare workers) during the pandemic, parents may choose to self-isolate from their children to protect them from the virus (Fitchel and Kaufman, 2020). In some cases, children of these healthcare workers may be left without the caregiver that typically engages in shared book reading with them.

Regardless of the situation, *video chat* technologies present an exciting opportunity for children to experience one-on-one interactions with caring adults (Ames et al., 2010; McClure and Barr, 2017). While parents have been long concerned with the effects of media exposure on young children, research suggests that video chat may encourage more interactive adult-child exchanges than other media-based activities such as playing solo games (Roseberry et al., 2014). Video chatting engages children for longer periods of time, for example, with long-distance family members than traditional phone calls (Ballagas et al., 2009), promoting social relationships with family and friends. Children have access to devices for video chatting at an early age. Indeed, 98% of children under eight now have access to a mobile device at home, and the average time children spend on mobile devices tripled between 2013 and 2017 (Rideout, 2017). Digital media is also entering the classroom: over half of early childhood teachers report using tablets in their classrooms at least once a week (Blackwell et al., 2015).

Research on toddlers’ learning from video chatting suggests that this technology may be effective for promoting literacy and language development because conversations can be *contingent* – adults’ responses can be temporally and topically related to children’s utterances (Troseth et al., 2006). Roseberry et al. (2014), for example, found that 2-year-olds learned novel words when taught over video chat but not when watching a prerecorded video of an adult teaching the word to another child. Crucially, the prerecorded video lacked well-timed, back-and-forth communication or contingency. Similarly, Myers et al. (2016) had 12–25-month-olds participate in six sessions in which they either video chatted with a researcher or watched a prerecorded video of a researcher on a tablet. Children demonstrated more synchronous behavior (e.g., waving when the experimenter waved) during video chat than when watching prerecorded videos. Children were also more likely to prefer the partner they interacted with to a new partner in the video chat condition than the prerecorded condition. Finally, older children (between 22 and 24 months) in the video chat condition performed significantly better on word learning tasks than children in the prerecorded condition.

Findings concerning how toddlers are affected by contingency may extend to older children, as well. For example, one study showed that 3-year-olds only passed a stringent test of verb learning when verbs were taught over a prerecorded video in addition to a live adult training session (Roseberry et al., 2009). Children were unsuccessful in passing the stringent test when they were taught through video alone. Other research has demonstrated that 4‐ and 5-year-olds comprehend an e-book better after reading with a parent than after viewing the e-book independently with audio narration, again suggesting that the contingent interactions that occur with an adult may promote learning (e.g., Dore et al., 2019). In the current study, we assess how preschoolers learn from being read a book over video chat. Given the known importance of dialogic reading, children may similarly benefit from the socially contingent interactions that occur over video chat. For many children, video chat may be a familiar and effective way to connect children with caring adults as reading partners, even if they are not physically present. We chose to examine preschoolers for several reasons. First, little research has explored learning over video chat with this age range, although they likely have the attention and social skills needed to have extensive, meaningful interactions over video chat (e.g., Tarasuik et al., 2011). Second, given that preschoolers benefit from contingent interactions during dialogic reading, video chat affords these interactions, even when adults and children are physically apart.

Although research suggests that video chat can help children’s language skills, having previous experience with this technology may be important for learning outcomes. Similar effects have been demonstrated with TV; Crawley et al. (2006), for example, found that 3‐ and 5-year-olds with previous exposure to *Blue’s Clues* are more likely to respond to characters’ questions in the show than children who did not previously watch the show. Similarly, Kirkorian and Choi (2017) found that toddlers who use more interactive media (apps and games) learn better from media in general, suggesting that experiences with interactivity may have shown them that media can be responsive and a reliable source of information. Increased experience with video chat may also help children understand that the partner on the screen can communicate with them and will respond to them in meaningful ways. Previous research on children’s learning from video chat reports no relationship between exposure to video chat and performance in lab-based studies (Myers et al., 2018; Strouse et al., 2018). However, children in these studies were younger than children in the current sample. By preschool, the degree to which children have had video chat experience may have an effect on their learning, with years of experience to help them understand the nature of video chat. Therefore, we asked parents in the current study how frequently their children video chatted. We expected to find a moderate effect of previous video chat use, such that children with more experience video chatting would benefit most from video chat book reading.

In the current study, we focus on two key skills that follow from book reading, story comprehension and vocabulary learning. The current study compares how dialogic reading practices over video chat affect children’s story comprehension and vocabulary learning. We focus on these outcomes because they are well-established benefits of storybook reading and dialogic reading practices (e.g., Whitehurst et al., 1988; Hargave and Sénéchal, 2000; Clarke et al., 2010; Montag et al., 2015). We tested comprehension to assess the extent to which children can comprehend a story *via* video chat, a prerequisite to any additional learning or other benefits of storybook reading. To ensure a stringent test, we used three measures of comprehension: an open-ended retell task in which children tell the story to the experimenter, an explicit comprehension task in which children are asked questions about events occurring in the story’s plot, and an implicit comprehension task in which children are asked questions assessing their ability to make inferences based on the story. Second, we tested vocabulary to assess the extent to which children can learn new vocabulary words *via* video chat. Again, we used three measures to ensure a robust test of this question. These were: a recognition task in which children had to link the vocabulary word to a related image from the book, a transfer task in which children had to link the vocabulary word to a novel image, and an expressive vocabulary task in which children had to provide the meanings of the vocabulary words.

To evaluate the possible benefits of video chat reading, different children were read to by a live experimenter, an experimenter over video chat, or an experimenter in a prerecorded video in a between-subject design. In addition to testing the effectiveness of video chat, these three conditions were chosen to assess the unique roles of (a) screen media and (b) contingency (See Table 1). The first aim of the study was to assess whether children could comprehend a book when read to through a digital screen. Children’s comprehension in the Live condition was compared to their comprehension in the Video Chat and Prerecorded conditions to assess whether children understood more from the story simply from interacting with a live reader, rather than a reader over a screen. The second aim of the study was to assess the role of contingency in children’s comprehension of the book. Both the Live and the Video chat conditions contained *contingency*; the reader could provide time-sensitive responses tailored to children’s individual behaviors. In contrast, the Prerecorded condition lacked these elements of true contingency and provided only predetermined responses to children. Children’s comprehension in the Live and Video chat conditions was compared to their comprehension in the Prerecorded condition to assess the role of contingency in children’s understanding of the book. The third aim of the study was to assess whether children’s responsiveness *during* book reading explained the effect of different reading formats (i.e., Live, Video chat, and Prerecorded) on their performance on outcome measures. Based on the literature, we hypothesized the following:

1. Children’s story comprehension and vocabulary learning in the Live and Video chat conditions will not differ, as both conditions include socially contingent partners.
2. Children’s story comprehension and vocabulary learning will be better in the Live and Video chat conditions than the Prerecorded condition, as the Prerecorded condition is not contingent.
3. Children will be more responsive to the reader (e.g., answer questions and respond to prompts) during book reading in the Live and Video chat conditions than in the Prerecorded condition.
4. Children’s responsiveness to questions and prompts used during book reading will be related to their story comprehension and vocabulary learning.

**Table 1:** Outline of condition affordances.

	Prerecorded	Video chat	Live
Dialogic reading prompts	√	√	√
Contingent	×	√	√
Non-mediated (not on a screen)	×	×	√

## Materials and Methods

### Design

We first randomly assigned participants to our three primary conditions (Prerecorded, Video chat, and Live) and conducted book reading and measures of comprehension and learning. We subsequently added a small sample of children who completed the comprehension and learning measures but were not exposed to the book as a control group for comparison.

### Participants

A total of 58 4-year-olds (31 girls, *M*_age_ = 52.70, *SD*_age_ = 4.04) were randomly assigned to the three primary conditions. Sixteen additional participants were tested but excluded due to failure to complete the procedure (*n* = 8), being out of age range (*n* = 1), having already read the book (*n* = 1), audio recording malfunction (*n* = 4), experimenter error (*n* = 1), or a diagnosed developmental delay (*n* = 1). All data were collected prior to the COVID-19 pandemic. Our sample was largely homogeneous; participants were predominately white (74% of children), middle-class (78% of primary caregivers held at least a bachelor’s degree), and spoke English as their primary language (100% of sample). Demographic information about the sample is provided in Table 2. Participants were recruited and the study was conducted at two separate sites. At one site, participants were recruited by telephone and email from databases of families willing to participate in research at laboratories based at a Mid-Atlantic University. At the second site, a Midwestern University, participants were recruited from local early childcare centers. As reading practices have been shown to differ across socioeconomic status (SES; Huebner, 2000), we assessed whether conditions differed by caregivers’ education, a core dimension of SES (Molborn et al., 2014). Two ANOVA’s revealed that primary caregivers’ [*F*(2,55) = 0.565, *p* = 0.571] and secondary caregivers’ [*F*(2,54) = 0.405, *p* = 0.669] education did not differ by condition.

**Table 2:** Demographic characteristics of sample by condition.

	Live	Video chat	Prerecorded	Control
Age in months (*SD*)	52.01 (3.01)	52.25 (4.42)	53.05 (4.40)	50.70 (2.32)
Site				
Site 1	11	16	15	9
Site 2	7	5	4	2
Gender				
Male	8	10	9	5
Female	10	11	10	6
Primary caregiver education				
Less than bachelor’s degree	3	4	6	4
Bachelor’s degree	3	6	4	2
Graduate degree	12	11	9	5
No response	0	0	0	0
Secondary caregiver education				
Less than bachelor’s degree	5	9	6	1
Bachelor’s degree	6	6	5	2
Graduate degree	7	5	8	8
No response	0	1	0	0
Race/ethnicity				
White	15	16	12	9
Black	1	1	1	0
Hispanic	0	0	1	0
Asian	1	2	2	1
Other/multiple races	1	0	1	1
No response	0	2	2	0

Experimental *N* = 58 for transfer vocabulary measure, 57 for receptive vocabulary measure, 56 for explicit comprehension measure, 54 for expressive vocabulary, 53 for page-by-page retell, and 52 for implicit comprehension; Control *N* = 11.

Note that sample sizes for each outcome measure differed slightly (*N* = 54 for expressive vocabulary, 57 for receptive vocabulary, 58 for transfer vocabulary, 56 for explicit comprehension, 53 for page-by-page retell, and 52 for implicit comprehension) due to issues with children’s cooperativeness. When children appeared uninterested in completing a particular task after multiple attempts to reengage them, the researcher moved onto the next task.

Participants in the control condition were 11 children (6 girls). Nine children were tested at site 1 and two were tested at site 2; this distribution was similar to the original sample (experimental: 72.4% at site 1, control: 81.8% at site 1). Primary and secondary caregiver education did not differ between the experimental and control conditions, [primary caregiver: *t*(67) = 0.673, *p* = 0.503; secondary caregiver: *t*(66) = −1.72, *p* = 0.089]. An independent-samples *t*-test revealed that children in the control condition (*M*_age_ = 50.70, *SD* = 2.32) were slightly younger than children in the experimental groups (*M*_age_ = 52.70, *SD* = 4.04), *t*(23.49) = 2.29, *p* = 0.032 (adjusting degrees of freedom in light of unequal variances in Levene’s test, *F* = 9.35, *p* = 0.003). However, notably we found no main effects of age or interactions between age and condition for any outcome measures *p*s > 0.265.

Parents provided written informed consent, and children provided verbal assent before entering the testing room. This project was approved by the University of Delaware Institutional Review Board and the Purdue University Review Board. All children received a certificate of appreciation and a sticker or a picture book after completing the study.

### Procedure

In the three experimental conditions, children saw two experimenters; one reader and one tester. Children never saw the reader until the actual reading session, to ensure children did not have any prior interactions with the reader beforehand. Across the three book reading conditions to which participants were randomly assigned, children were read the same book, *The Busy Beaver*, by Nicholas Oldland. This commercially available book was engaging for children of a similar age and demographic in previous research (Dore et al., 2018). Some of the words in the story were replaced with new words to make the vocabulary more challenging for 4-year-olds. Specifically, *forest*, *moose*, *chewed*, and *built* were replaced with *woodland*, *caribou*, *gnawed*, and *constructed*. These words, as well as additional target vocabulary words, were chosen because they were unlikely to be known by children of this age group (Dale and O’Rourke, 1981). Children in all three experimental conditions were read to by the same two female experimenters, one at each site. Furthermore, across all three experimental conditions, the reader used the same 10 scripted dialogic reading prompts during book reading. These prompts, adapted from the CROWD strategy (Whitehurst et al., 1988), included *recall* prompts (i.e., “What looks different now?”), *open-ended* prompts (i.e., “How do you think the birds felt now that they have a new home?”), *Wh*-prompts, (i.e., “What do you think the beaver’s going to do?”), and *distancing* prompts (i.e., “Have you ever gotten a booboo? What happened?”); see Table 3 for a full list of prompts used during book reading. In the Live and Video chat conditions, readers gave the children personalized feedback, based on children’s responses to prompts. Rather than providing children with more content than in the Prerecorded condition, the feedback in these conditions functioned to expand children’s responses or correct their answers (Table 4). The reader often repeated what children said, expanded on their response, and prompted them to continue to respond. In the Prerecorded condition, however, the readers’ feedback was scripted and did not vary based on children’s responses. Book reading took 7 min and 46 s on average and did not differ by condition, *F*(2,51) = 0.368, *p* = 0.694.

**Table 3:** Questions posed during book reading.

Question type	Questions
*Warm up*	What’s your favorite color?
	What do you see on the cover?
	Are you ready to find out what happens in the story?
*Dialogic reading questions*	Why do you think he (the beaver) thought the caribou’s leg was a tree?^*^
	Do you see something else that happened when the tree was falling?^*^
	Have you ever gotten a booboo? What happened?
	What do you think the beaver’s going to do?^*^
	How do you think the birds feel now that they have a new home?^*^
	Have you ever had to apologize to one of your friends? What happened?
	What looks different now?^*^

* Included as book-relevant questions for accurate response coding.

**Table 4:** Examples of reader’s feedback to children during reading.

Condition	Response Example
*Live*	*R*: What looks different now? *C*: The beaver’s swimming. *R*: He’s swimming? Anything else? *C*: He’s building. *R:* He’s building, yes. It all looks cleaner, huh?
*Video chat*	*R*: What looks different now? *C*: Yup. *R*: Huh? *C:* Yup. *R*: What looks different in the story? C: The house. *R:* The house looks better now? And it’s a little cleaner and there are no more trees anywhere? *C*: Yeah.
*Prerecorded*	*R*: What looks different now? *C:* No response. *R:* That’s right! He cleaned up his mess. Now there are no more trees and branches anywhere and they all look happier.

R, reader; C, child.

In the *Live* condition (*n* = 18), a first experimenter (i.e., tester) brought the child into the testing room and had them sit down at a table. A second experimenter (i.e., the reader) sat in the testing room across from the child in a second chair. The tester introduced the child to the reader, telling the child, “My friend is going to read you a story today!” The tester left the room during book reading. The reader greeted the child by name and introduced herself. The reader asked the child an opening question (i.e., “What’s your favorite color?”) and responded appropriately to the child (i.e., “I like [color child previously stated], too!”). The reader then held up the storybook and introduced the story to the child (i.e., “Today, I’m going to read you a story. The name of the story is *The Busy Beaver*”). The reader asked what the child saw on the cover of the book and provided a neutral comment to the child’s response. The reader also asked the child whether they were ready to see what happens in the story. After the initial warm up was complete, the reader read *The Busy Beaver* to the child, pausing the reading to use prompts and questions to encourage the child to talk about the book. After the book was completed, the reader left the room and the tester returned.

The procedure in the *Video Chat* (*n* = 21) condition was identical to the Live condition, except that the reader interacted with the child solely through FaceTime video chatting technology. The tester brought the child into the testing room, where the child was instructed to sit at the table. The tester angled an iPad tablet in front of the child, so that the camera on the tablet captured the child’s face. The tester then told the child, “My friend is going to read you a story today!” and then proceeded to call the reader over FaceTime. Once the reader answered the FaceTime call, she followed the same procedure as in the Live condition, beginning with a greeting and warm up and then reading the story and stopping to prompt the child and ask questions about the story. The tester remained in the room with the child to resolve any technical issues but sat behind the child during reading and did not interact with the reader or pay overt attention to the reading activity. After reading, the tester turned off the tablet and sat down across from the participant.

In the *Prerecorded* (*n* = 19) condition, children were also led into the testing room by the tester, placed in front of the iPad, and told, “My friend is going to read you a story today!” Instead of calling the reader over FaceTime, the tester turned on the tablet to reveal a prerecorded video of the reader. The tester remained in the room with the child to resolve any technical issues but sat behind the child during reading and did not pay overt attention to the reading activity. Prerecorded videos were created for each site to match the average reader word count and reading time of the first four live and first four video chat reading sessions. Specifically, the videos created at each university had word counts of 313 (8 min long) and 305 (9 min long), reflecting the average of the four video chat and live reading sessions at each site. This prerecorded video was *pseudo-contingent* in nature; the reader posed questions to the child during the story and paused for a set period of time (on average, 6.28 s after each question, *SD* = 2.78) as if to wait for a child response. Then, the reader provided the same generic feedback to the child’s response, regardless of the presence or the accuracy of the response. For example, after asking “What’s your favorite color?”, the reader always waited for a period of time and then responded, “I like that color, too!” After reading, the tester turned off the tablet and sat down across from the participant.

In the *Control* condition, children completed the outcome measures prior to reading the storybook. For each task, children were given the same instructions as children in the experimental conditions. However, testers gave additional emphasis on “doing your best” and mentioned that “these games might seem a bit silly” to ensure that children would not become frustrated by answering questions about a book they had not yet read. After completing the tasks, children watched the prerecorded video on the tablet.

### Outcome Measures

All children completed tasks in the same order: (1) expressive vocabulary, (2) receptive vocabulary, (3) transfer vocabulary, (4) page-by-page retell, (5) explicit comprehension, and (6) implicit comprehension. Tasks were always presented in the same sequential order so that earlier tasks would not provide information that could influence children’s later responses (e.g., the comprehension questions could provide information about the book that could be used to complete the page-by-page retell).

#### Expressive Vocabulary

The expressive measure was adapted from the New Word Definition Test–Modified (Hadley et al., 2015). Children were asked for the meanings of 10 vocabulary words from the book (i.e., *beaver*, *dam*, *felled*, *leaky*, *homeless*, *careless*, *caribou*, *construct*, *woodland*, and *gnaw*). Although the words appeared in the book, they were never explicitly taught to children, as research suggests that caregivers do not typically teach vocabulary during shared book reading (Evans et al., 2011). Additionally, research suggests that preschoolers can learn vocabulary words that are repeated during book reading, even in the absence of word definitions (O’Fallon et al., 2020). For nouns, children were asked, for example, “What is a dam?” and then, “Can you tell me or show me anything else about a dam?” For verbs, children were asked, for example, “What is gnawing?” and “Can you tell me or show me anything else about gnawing?” Testers gave children neutral feedback regardless of their accuracy, e.g., “You’re working so hard!” Prior to beginning the test words, children responded to two practice words (*drinking* and *tree*) to ensure that they understood the task. In the middle of the task, children were asked an additional practice word, *hat*, to ensure that they were responding attentively and to encourage them with an easier question. Responses to test words were coded for each information unit the child provided. We coded for eight information unit categories: perceptual qualities, functional information, part/whole, synonyms, antonyms, gestures, meaningful context, and basic context. Children’s verbal responses and relevant gestures were considered when coding responses. For example, if children gestured to represent hammering in response to *construct*, they received a point. Children received one point for each information unit provided from the first seven categories and half of a point for giving basic context of a word (e.g., “He was *constructing* a dam.”). To examine reliability for the expressive task, 20% of the participants (*n* = 11) were randomly selected and double-coded by a second coder. Reliability was high, Kappa = 0.814.

#### Receptive Vocabulary

Children were tested on the same 10 vocabulary words in the expressive task. However, children demonstrated their receptive knowledge of words instead of providing productive responses. For each word, children were shown images on two cards taken directly from the story and were asked to identify the image representing the target word. For example, children were shown images of the beaver and the caribou and asked, “Can you show me the beaver?” Foil images in the receptive task were all images from the book that were perceptually comparable to the image representing the target word. For example, for the word *beaver*, both choices showed images of a single character; one image was a bear standing upright on a white background and the other image was a beaver standing upright on a white background. For the word *homeless*, children saw two options, both containing three birds. In the target option, the birds held sacks and walked on the ground. The children had previously seen this image in the book when the birds were described as *homeless*. In the foil option, the three birds were shown in their nest, representing birds in their home. Children received one point for a correct answer and zero points for an incorrect answer.

#### Transfer Vocabulary

In this stringent test of word knowledge, children viewed four photographs from real-world contexts not represented in the book. Children were tested on the same 10 words as in the expressive and receptive tasks. As in Dore et al. (2019), foil selection was guided by research on lexical development (e.g., Golinkoff et al., 1995) and included three types of foils: (1) *thematic* (frequently found in the same event or situation, e.g., a forest for the word *beaver*); (2) *conceptual* (shares a common category, e.g., animal, as in a panther for *beaver*); and (3) *phonological* (rhymes with the target word, e.g., fever for *beaver*). In the task, children must generalize beyond the book’s picture to a new exemplar and choose between meaningfully-related options. Children were instructed to point to the target word and received one point for selecting the target and zero points for selecting any of the three foils.

#### Page-by-Page Retell

Adapted from Dore et al. (2018), the researcher showed children printed screenshots of the book’s pages with the text removed and asked children to retell what happened on each page of the story. Instructions were revised slightly for the control condition; rather than being asked to retell the story, children were asked, “On each page, can you tell me what you think is happening?” Across conditions, on the first page, researchers would say “I’ll get you started… There once was a….” If needed, children were given encouraging comments in a set order (e.g., “What happened here?” or “Do you remember anything else?”). If children pointed, were vague, or said “this” or “that,” researchers would prompt them to verbalize (e.g., “Who?” and “What is that?”). Researchers did not include any specific information in their prompts or give children any feedback. Responses were coded by counting how many of a predetermined set of possible elements children recalled from the story, based on coding established in Dore et al. (2018). To examine inter-coder reliability, a second trained coder, blind to the original coding, coded a randomly selected 20% of the data. For each of the identified possible elements children could retell, agreement between the two coders was examined. After removing 65 of the identified elements that were never recalled by any of the children, average agreement for the elements children recalled was 94.12%. Where there were disagreements, the original coder’s decision was retained. Kappa was also calculated with all possible elements included. Reliability was high, Kappa = 0.858.

#### Explicit Comprehension Questions

In the explicit comprehension task (adapted from Dore et al., 2018), children were asked five multiple-choice comprehension questions about the content of the story with two response options, such as “How did the beaver get better at saying ‘I’m sorry?’ (A) He read a book about it. (B) He practiced in the mirror.” Response options were read and also represented visually by showing children two cards with illustrations from the story. Questions were developed to assess children’s understanding of basic story events. Children could not realistically answer these questions solely from looking at the photos. In the previous example, for instance, the beaver both read a book and practiced something in the mirror during the story. Beyond identifying pictures from the story, children had to assess which picture from the story accurately answered the question. If needed, questions were repeated to make sure that the child understood the question and the response options. Children who were unsure or reluctant to provide an answer were told to give their best guess.

#### Implicit Comprehension Questions

The implicit task, adapted from Paris and Paris (2001), assessed children’s ability to make appropriate inferences about photos using information from pages in the book. Children were asked five questions that focused on making inferences about characters’ feelings, causation, dialogue between characters, predictions, and overall theme. For example, children were shown a photo of a bear with a bandage on his head and asked, “Tell me what the bear is feeling in this picture. Why do you think so?” Children received a score of 2 for responses that indicated an inference that drew on events from multiple pages in the book, a 1 for an appropriate inference that was limited to events on the page, and a 0 for an inappropriate inference or response. Scoring was based on a coding scheme established in Paris and Paris (2001). To examine inter-coder reliability, a second trained coder, blind to the original coding, coded a randomly selected 20% of the data. Reliability was high, Kappa = 0.848.

### Responsiveness Without Regard to Accuracy

Children’s responsiveness to questions posed during book reading was coded by a trained research assistant and the first two authors. Coders watched videos of the reading sessions and noted whether a child provided a response for each of the 10 questions during book reading. Any meaningful verbal or nonverbal (e.g., a head nod in response to a yes/no question) behavior was coded as a response. For each of the 10 questions, children received either a 1 for a response or a 0 for no response. As this measure was focused purely on whether children gave a response, accuracy was not considered. To examine inter-coder reliability, a second trained coder, blind to the original coding, coded a randomly selected 20% of the data. Reliability was perfect, Kappa = 1.

### Accurate Responding

Next, children’s accurate responding to book-relevant questions was coded. A coding scheme was developed to give children points for answering the question with accurate information. Only questions that were specific to the book plot were included (see Table 3). For example, for the question “Why do you think the beaver thought the caribou’s leg was a tree?”, children received points for mentioning “it’s brown,” “it’s skinny,” or “it looks like a tree.” Children received one point for each unit of accurate information provided. In the previous example, if the child responded “because it’s brown and skinny,” the child would receive two points for the question. To examine inter-coder reliability, a second trained coder, blind to the original coding, coded a randomly selected 20% of the data. Reliability was substantial, Kappa = 0.79.

## Results

Results from the six comprehensions and vocabulary measures are presented first as the primary aim of the study was to assess how different book formats affected children’s reading comprehension. This is followed by results of children’s responsiveness to the story during book reading. Diagnostic analyses identified one outlier in the receptive vocabulary task (low score of 2), one outlier on the expressive vocabulary task (high score of 12), and two outliers in the implicit comprehension task (low scores of 1 and 2). These outliers, which were defined as more than 1.5 times the interquartile range above third quartile or below the first quartile, were excluded in analyses conducted on their respective outcome measures. Separate independent-samples *t*-tests were conducted to assess differences on the outcome measures between the two testing sites. One difference emerged, such that children at site 1 (*M* = 6.69, *SD* = 1.64) scored significantly higher on the transfer vocabulary test than children at site 2 (*M* = 5.25, *SD* = 1.95), *t*(56) = 2.83, *p* = 0.006. Thus, an ANOVA was run to test for an interaction between condition and site for the transfer task. This model was not significant, *p* = 0.846. No other differences on the remaining outcome measures between the two testing sites were observed (*p*s > 0.236). Non-parametric tests were conducted for the receptive vocabulary and explicit comprehension tasks as scores on these measures were not normally distributed.

### Story Comprehension and Vocabulary Learning

**Figure 1:**
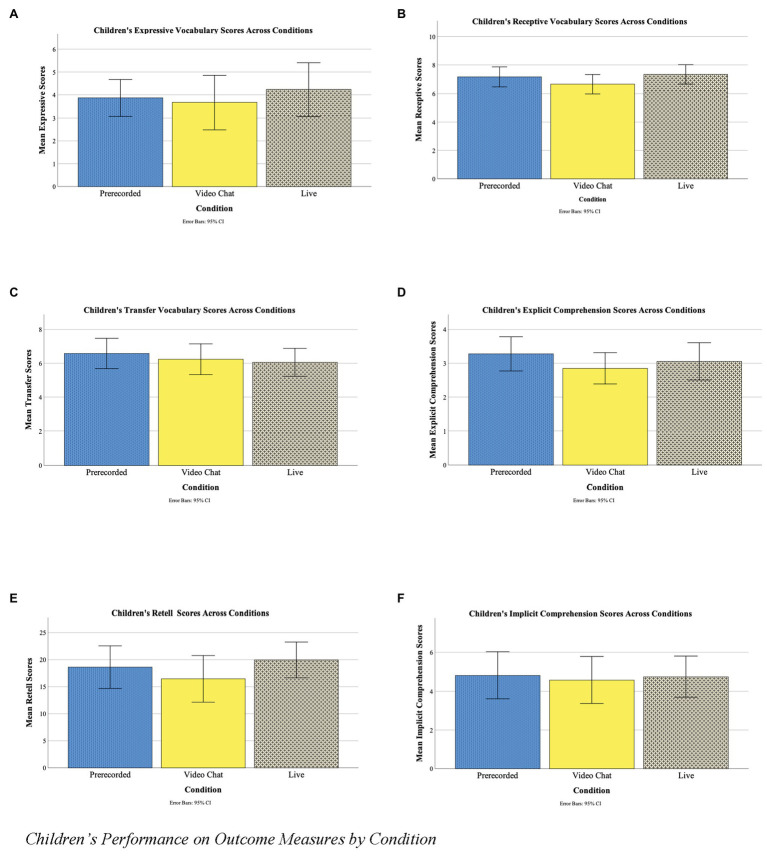
Children’s performance on outcome measures by condition. **(A)** Children’s expressive vocabulary scores across conditions. **(B)** Children’s receptive vocabulary scores across conditions. **(C)** Children’s transfer vocabulary scores across conditions. **(D)** Children’s explicit comprehension scores across conditions. **(E)** Children’s retell scores across conditions. **(F)** Children’s implicit comprehension scores across conditions.

Next, we assessed whether the experimental conditions differed on the six outcome measures. To test our first two research questions, (1) whether children learned equally well through video chat and live book reading and (2) whether contingency in book reading affected children’s reading comprehension and vocabulary, separate ANOVAs and nonparametric Kruskal-Wallis tests were conducted. Note that the control condition is presented separately from analyses comparing the three conditions, as this data was collected after the original sample and was *post hoc* in nature. Additionally, the control group is presented separately to maximize statistical power and avoid comparing unequal sample sizes in the main analyses. Children’s performance on each outcome measure was compared across conditions. No main effects of condition were found for the expressive vocabulary, [*F*(2,51) = 0.323, *p* = 0.725, *d* = 0.217, *n* = 54], transfer vocabulary [*F*(2,57) = 0.382, *p* = 0.684, *d* = 0.073, *n* = 58], implicit comprehension [*F*(2,49) = 0.054, *p* = 0.948, *d* = 0.090, *n* = 52], page-by-page retell task [*F*(2,50) = 0.908, *p* = 0.410, *d* = 0.372, *n* = 53], explicit comprehension [*X*^2^ (2) = 1.58, *p* = 0.453, *d* = 0.204, *n* = 56], or receptive vocabulary [*X*^2^(58) = 2.54, *p* = 0.281, *d* = 0.316, *n* = 57] tasks. Children performed similarly on comprehension and vocabulary measures across conditions, suggesting that they were not affected by the differing levels of contingency in book reading sessions (see Table 5 and Figure 1).

**Table 5:** Descriptive results for vocabulary and comprehension measures by condition.

	Expressive vocabulary	Receptive vocabulary	Transfer vocabulary	Explicit comprehension	Implicit comprehension	Page-by-page retell
Possible range	0–85	0–10	0–10	0–5	0–10	0–133
*Live*	4.24 (2.27)	7.33 (1.37)	6.06 (1.66)	3.06 (1.11)	4.75 (1.98)	19.94 (6.45)
*Video chat*	3.67 (2.38)	6.65 (1.42)	6.24 (2.00)	2.85 (0.99)	4.58 (2.50)	16.44 (8.68)
*Prerecorded*	3.87 (1.66)	7.16 (1.46)	6.58 (1.86)	3.28 (1.02)	4.82 (2.35)	18.61 (7.93)

Experimental *N* = 58 for transfer vocabulary measure, 57 for receptive vocabulary measure, 56 for explicit comprehension measure, 54 for expressive vocabulary, 53 for page-by-page retell, and 52 for implicit comprehension.

To test whether younger children (i.e., closer to 4 years) were more affected by contingency (i.e., performing better in the Live and Video chat conditions) than older children (i.e., closer to 4.9 years), analyses were also conducted to examine whether children’s age moderated the effect of condition on outcome measures. Separate two-way ANOVAs tested whether there were any interactions between age (entered as a continuous variable) and condition for outcome measures. Models were not significant for any of the tasks, *p*s > 0.265. Based on their performance on comprehension and vocabulary tasks, older and younger children learned similarly across different book formats.

Preliminary analyses indicated that children were at floor for some words on the expressive vocabulary task. On average, children scored less than 0.10 point for *felled* (*M* = 0.065, *SD* = 0.22), *caribou* (*M* = 0.083, *SD* = 0.27), and *gnaw* (*M* = 0.09, *SD* = 0.35). Thus, an ANOVA was conducted to compare children’s performance on the expressive task by condition excluding these three words. As with the initial model, no condition differences were observed after excluding the three challenging words, *F*(2,52) = 0.230, *p* = 0.795.

Analyses were also conducted to examine whether parent-reported video chat use moderated the effect of condition on outcome measures. Separate two-way ANOVAs tested whether there were any interactions between video chat use and condition for outcome measures. Models were not significant for any of the tasks, *p*s > 0.305.

### Responsiveness During Reading Sessions

Next, we tested our third research question, whether children were more responsive to the scripted prompts in the Live and Video chat conditions than the Prerecorded condition. Children’s responsiveness during book reading was analyzed based on coding any meaningful verbal or nonverbal response to the reader’s question or prompt. As responsiveness was not normally distributed, non-parametric tests were employed. A Kruskal-Wallis test revealed that the frequency with which children responded to the reader’s prompts during book reading differed by condition, *X*^2^(2) = 10.48, *p* = 0.005, with a median of 5.67 (*SD* = 4.03) for Prerecorded, 9.19 (*SD* = 1.91) for Video chat, and 9.27 (*SD* = 0.80) for Live. Children in the Video chat (*p* = 0.001) and Live (*p* = 0.038) conditions responded more to the reader’s questions during book reading than those in the Prerecorded condition. There was no difference in children’s responsiveness between the Video chat and Live conditions, *p* = 0.381.

To test our fourth research question, Pearson and Spearman correlations were conducted to assess relationships between children’s overall responsiveness during reading and performance on outcome measures. No significant correlations were observed for the expressive vocabulary (*r* = 0.008, *p* = 0.957), transfer (*r* = −0.120, *p* = 0.400), implicit comprehension (*r* = 0.229, *p* = 0.126), retell (*r* = 0.119, *p* = 0.388), explicit comprehension (*r*_s_ = −0.052, *p* = 0.718), or receptive (*r*_s_ = −0.166, *p* = 0.248) tasks. Separate two-way ANOVAs were run to test whether responsiveness was differentially related to children’s outcomes across conditions. Models were not significant for any of the measures (*p*s > 0.216), suggesting that although children’s responsiveness differed by condition, their responsiveness did not moderate the effect of condition on their comprehension or vocabulary scores.

Next, children’s accurate responding to prompts during book reading was analyzed. A Kruskal-Wallis test found no differences between conditions on accurate responding, *X*^2^(2) = 1.37, *p* = 0.504. Pearson and Spearman correlations were conducted to compare children’s performance on each task and their total number of accurate responses during reading. Children who provided more accurate responses to questions relevant to the book during book reading performed better on the expressive vocabulary (*r* = 0.472, *p* = 0.001), implicit (*r* = 0.499, *p* < 0.001), retell (*r* = 0.429, *p* = 0.002), explicit comprehension (*r*_s_ = 0.300, *p* = 0.029), receptive (*r*_s_ = 0.276, *p* = 0.044), and marginally, transfer (*r* = 0.254, *p* = 0.061) tasks. Based on these correlations, separate two-way ANOVAs were conducted to test for interactions between condition and children’s accurate responding predicting performance on outcome measures. Models were not significant for the receptive (*p* = 0.174), transfer (*p* = 0.237), or implicit comprehension (*p* = 0.144) tasks. The model for the page-by-page retell task was significant, *F*(23) = 1.98, *p* = 0.047. A marginal interaction was observed between children’s accurate responding and condition for the retell measure, *F*(9,49) = 2.04, *p* = 0.075. This interaction was further explored through correlations. Children’s accurate responding during book reading prompts was positively related to the retell measure in the Video Chat (*r* = 0.666, *p* = 0.003) and in the Live conditions (*r* = 0.523, *p* = 0.045) but not in the Prerecorded condition, *r* = 0.072, *p* = 0.782.

### Results From the Control Condition

Lastly, analyses were conducted to assess whether children in the three experimental groups outperformed children in the control condition, who had not read the book. Results revealed a similar pattern across measures (see Table 6). Children in the experimental conditions scored significantly higher than children in the control condition on the receptive vocabulary test (*U* = 497.50, *z* = 2.97, *p* = 0.003) and the explicit comprehension test, *U* = 446.00, *z* = 2.28, *p* = 0.022. Independent-samples *t*-tests were conducted to compare children’s performance on the expressive vocabulary, transfer vocabulary, retell, and implicit measures. Children in the experimental conditions outperformed children in the control condition on the transfer vocabulary [*t*(67) = −2.07, *p* = 0.043] and retell [*t*(62) = 3.91, *p* < 0.001] tasks and marginally outperformed the control condition on the implicit comprehension task, *t*(63) = 1.74, *p* = 0.087. However, children in the experimental conditions did not score significantly higher on the expressive vocabulary task than children in the control condition, *t*(64) = 1.54, *p* = 0.129.

**Table 6:** Descriptive results for experimental and control conditions; Mean (standard deviation).

Measure	Experimental conditions	Control condition	Effect size(Cohen’s *d*)
*Expressive vocabulary*	4.06 (2.34)	2.82 (2.99)	0.46
*Receptive vocabulary*	6.84 (1.80)^*^	5.27 (1.42)	0.97
*Transfer vocabulary*	6.16 (2.00)^*^	4.82 (1.78)	0.71
*Explicit comprehension*	3.00 (1.10)^*^	2.09 (1.22)	0.78
*Implicit comprehension*	4.54 (2.39)^†^	3.18 (2.14)	0.60
*Page-by-page retell*	18.30 (7.75)^**^	8.64 (5.77)	1.41

† Indicates *p* < 0.10.

* Indicates *p* < 0.05.

** Indicates *p* < 0.001.

## Discussion

The current study tested whether preschoolers would comprehend a book differently if read to by a live experimenter, an experimenter on video chat, or an experimenter on a prerecorded video. Results revealed that children responded more to dialogic prompts and questions posed during book reading in the contingent conditions (i.e., Live and Video chat) than in the pseudo-contingent condition (i.e., Prerecorded). Despite this difference, results ultimately suggest that 4-year-old children comprehended a storybook similarly regardless of book reading format. Additionally, neither children’s age nor previous video chat use affected how children comprehended the book in different formats.

Importantly, children’s comprehension did not differ between the *Video chat* and *Live* conditions. Although we expected children to comprehend major story elements in both the Video chat and Live conditions as both were conducted by contingent social partners, children might be expected to comprehend more from reading with a live adult who might provide more social cues to children during video chatting. However, the 4-year-olds in this study comprehended just as much from the story when they were read to over video chat as when read to by a live experimenter. One reason children may have comprehended the story equally well is the prevalence of video chat in children’s lives – even before the COVID-19 pandemic, participants had experience with video chatting. In the current sample, parents were asked about their child’s use of video chat technologies (e.g., Skype or FaceTime), and out of 58 parents, 51 reported that their child had video chatted in the past. Considering that at least 87.9% of children in the current study had already used video chat, these children may have been well-accustomed to interacting with digital partners over screens, leading to equal comprehension across the Video chat and Live conditions. Contrary to our expectations, children’s previous experience with video chat did not moderate the effect of condition on any of the outcome measures. Other research similarly reports a null relationship between children’s prior experience with video chatting and their performance in lab-based video studies (Myers et al., 2018; Strouse et al., 2018). Perhaps the contingent interactions in video chat conversations are so similar to live, in-person conversations that children do not need extensive experience with video chatting to learn from it. Although the children in the current study were familiar with video chatting, even children with less experience may learn from the book reading activity.

Children’s comparable performance in the Prerecorded condition to the other two conditions was still somewhat surprising in light of the literature on the role of contingency in children’s learning (Troseth et al., 2006; Lauricella et al., 2010; Roseberry et al., 2014). This research would suggest that the Video chat and Live conditions, including the element of contingent interactions, would outperform the Prerecorded condition, which lacked true contingency. Although we hypothesized that the 4-year-olds in our study would similarly struggle to learn from a prerecorded video and benefit from *social* contingency, previous work was mostly conducted with toddlers. By the preschool years, children’s learning may not be as sensitive to contingency. Indeed, several studies suggest that *touch screen* contingency (e.g., requiring children to touch the screen to reveal a hidden object) may actually be detrimental for older children’s learning. For example, some studies found that while children around 2 years old learn better from a contingent touchscreen interaction, children closer to 3 years of age learn equally well or even better from watching a non-contingent video, devoid of touchscreen interaction (Choi and Kirkorian, 2016; Kirkorian et al., 2016). A similar study found that preschoolers learned better from watching a recording of game play than from playing the digital game themselves, possibly because cognitive load is too high during play for children to encode new information (Schroeder and Kirkorian, 2016; see also Aladé et al., 2016). Although there are fewer studies of social contingency with preschool-aged children, it is possible that learning from social contingency and touch screen contingency may pattern similarly, and in the current study, 4-year-olds no longer needed true social contingency to learn from the story. One study mimicking social contingency compared 3‐ and 5-year-olds with previous exposure to *Blue’s Clues*, a TV show with elements of pseudo-contingency, to children who had not been exposed to the show media (Crawley et al., 2006). Children who had previously watched *Blue’s Clues* responded more to prompts both during a *Blue’s Clues* episode and during a new TV show than children who had not been previously exposed to *Blue’s Clues*, suggesting that children are responsive to the pseudo-contingent style in the domain of social communication. Beyond comprehending the story, children in the Prerecorded condition also performed just as well as children in the other two conditions on measures of vocabulary. Although contingent conversations may be best for early language development (Hirsh-Pasek et al., 2015a), some research suggests children can learn vocabulary words even when listening to a book read a single time verbatim (Sénéchal and Cornell, 1993). Perhaps by 4 years of age, children can gain some vocabulary knowledge even through passively listening to a story.

Similarly, by 4 or 5 years of age, children may not be as sensitive to book formats in general, in line with a previous study showing that, unlike 3-year-olds, 5-year-olds did not demonstrate decreased comprehension from the distracting features in that study’s console book, the predecessor of e-books (Parish-Morris et al., 2013). Parish-Morris et al. (2013) suggested that the 5-years-olds in their study comprehended the basic narrative structure from e-books, even when hotspots and sound effects disrupt the 3-year-olds’ comprehension of the book.

As we did not originally include any pretest measures, we tested a sample of children on the measures *before* reading them the book to create a control group. These results confirmed that children in all three experimental conditions (i.e., Live, Video chat, and Prerecorded) indeed learned from hearing the book. Children gained significant plot information from the story, as demonstrated in the explicit comprehension and retell tasks, and learned vocabulary words, as seen in the receptive and transfer tasks. Crucially, these vocabulary words were never taught explicitly in the book. Our results align with previous research (O’Fallon et al., 2020), which suggests that young children can learn new vocabulary words during book reading without explicit instruction. However, although children in the experimental conditions scored higher than children in the control condition on the expressive vocabulary measure, this difference did not reach traditional levels of statistical significance. This finding was somewhat inconsistent with previous research, as studies commonly find that dialogic reading improves children’s *expressive* vocabulary, but not necessarily children’s *receptive* vocabulary (e.g., Whitehurst et al., 1988, 1994; Lonigan and Whitehurst, 1998; Hargave and Sénéchal, 2000). Additionally, research using a similar expressive vocabulary task found that after a book reading intervention, preschoolers did show significant improvements in their knowledge of target vocabulary words (Toub et al., 2018), suggesting that the task was not beyond children’s ability level in the current study. However, reading occurred over multiple sessions in the prior studies that found positive effects on expressive vocabulary. Previous research suggests children struggle to perform on expressive vocabulary tasks after a single book reading session (Sénéchal and Cornell, 1993). In the current study, although children identified vocabulary words from corresponding photos, a single book reading session may not have been sufficient for them to talk about the meanings of the new words. When looking across all outcome measures, results from the control condition suggest that children comprehended the story and gained receptive vocabulary knowledge through reading the story across all three book reading conditions.

Despite a lack of differences between our experimental conditions on outcome measures, children were overall more responsive in the Live and Video chat conditions than the Prerecorded condition, indicating that they were sensitive to the fact that these interactions contained contingency that was lacking in the Prerecorded condition. Yet, across conditions, children’s responsiveness did not relate to their performance on the comprehension and vocabulary measures. However, analyzing the *content* of children’s responses during book reading revealed an interesting interaction. Children’s accurate responding during book reading prompts was marginally positively related to the retell measure in the Video chat and Live conditions, but not in the Prerecorded condition. One explanation for this finding is that in the Video chat and Live conditions, the reader could tailor her reactions to individual children’s responses, adding relevant information and expanding on children’s comments directly. Children in the Prerecorded condition had the opportunity to respond to questions and prompts during book reading, but the reader could not give the personalized feedback that was possible in the other two conditions. Beyond simply giving children the opportunity to respond to a question, the readers in the Live and Video chat conditions also asked children for further clarifications of their responses and asked children to give additional information. This additional feedback may have been key for promoting children’s comprehension of the story. As a consequence, some children engaged in back-and-forth communication with the reader in their responses (See Table 4), adding details to their answers. The Live and Video chat readers’ comments may have encouraged children to further clarify and expand on their responses, helping keep children focused on key story elements. Future research on dialogic reading should focus on how readers’ feedback to children affects their reading comprehension and learning from the story in both digital and live contexts.

Importantly, these results reflect 4-year-olds’ learning from the book irrespective of any adult co-viewing behaviors. Although children did not seem to notice the testers’ lack of overt attention to the tablet during book reading sessions, it is possible that it affected their reading experience. Some research has shown that 30-month-olds learned novel words best when watching a contingent video with a parent who modeled responsiveness to the video than when the parent was out of the child’s view (Strouse et al., 2018). In fact, even having a parent co-view a prerecorded video aided children’s learning. In the current study, *preschoolers* responded more frequently to the contingent video chat reader than to the pseudo-contingent, prerecorded reader, suggesting that even without an adult co-viewer, by 4 years of age, children were able to differentiate between the video chat and prerecorded videos. Regardless of their responses, preschoolers learned from both video formats, without the presence of an attentive adult co-viewer.

### Other Potential Benefits of Reading With a Live Adult

The findings of this study have several practical implications. First, our findings suggest that 4-year-olds can glean story details from simply watching a prerecorded video of a storybook reading. Although it might be tempting to conclude that watching TV or video content would be comparable to live interactive book reading, it is possible that commercially-available videos (e.g., TV, DVDs, and YouTube) would not yield the same effects. Specifically, because of the experimental nature of this study, the Prerecorded condition was explicitly designed to be as closely matched to the other two contingent conditions as possible. The video focused exclusively on the reader, who sat in a room with bare walls. Typical TV programs are likely to include more engaging features (e.g., animation, sound effects, and scene transitions) that could detract from children’s attention to the story (see Bus et al., 2015, for an example in the domain of e-books). Furthermore, although some TV shows and other video content include characters directly addressing the viewer, many do not. The current findings suggest that, in line with some prior research (Krcmar and Cingel, 2017), having video characters directly address viewers may facilitate comprehension (see also Crawley et al., 2006). Storybooks read over video that lack this feature may not be as effective.

Additionally, even if children in this age range can comprehend stories from videos, a live adult is required for many of the positive outcomes traditionally associated with shared book reading. For example, research suggests that contingent, back-and-forth communication is best for promoting children’s language skills in general, at least, for younger children (Hirsh-Pasek et al., 2015a; Romeo et al., 2018; Merz et al., 2019). Children can practice back-and-forth conversation during book reading both in person and over video chat by responding to adults’ dialogic reading questions and receiving feedback catered specifically to their response but not when watching a prerecorded video. Additionally, during storybook reading, children gain print knowledge (Leseman and deJong, 1998; Justice et al., 2008; Korat, et al., 2009), learn to identify the relationship between printed text and oral words, and begin to understand the function of printed text (Mason, 1980; Hiebert, 1981; Justice and Ezell, 2001). The current study did not address whether children can also gain these important skills in a prerecorded book reading format. Another potential advantage over the prerecorded format relates to the emotional experience of shared book reading. Preliminary results from an ongoing study (Avelar et al., in preparation) suggest that reading with a parent is a different emotional experience than reading an e-book independently, with shared reading associated with greater physiological arousal and more positive emotion in 4-year-olds (Dore et al., 2019), a difference that may not extend to watching a video of prerecorded book reading.

### Implications for Families During COVID-19 and Beyond

Although prior research shows that reading with an adult in person has widespread advantages for children, the results of the current study suggest that even when they are physically apart, adults can support preschoolers’ reading comprehension with video chat. This finding has promising implications for many families. Primarily, these results suggest that during the current COVID-19 crisis and any similar stay-at-home orders in the future, 4-year-olds *can learn* when read to over video chat. When allowing their children to read a book over video chat with a distant family member, parents can feel confident that children are likely comprehending the story and may even be learning new vocabulary words. Without knowing how long the current pandemic will last or whether we will face another wave of the pandemic in the future, it is imperative for parents to be armed with knowledge of virtual activities that are beneficial for their young children. Furthermore, preschoolers with separated or divorced parents living in separate homes, incarcerated parents, and parents living in other countries could all potentially benefit from reading with parents through video chat, both during the pandemic and in typical times. For example, programs have been developed to help incarcerated parents record videos of themselves reading to their children (Barker, 2019). The current study suggests that by age 4, children may comprehend these stories. Although more research is needed, children may experience benefits in other domains (language skills and emotional bonding) from reading over video chat.

Additionally, children from disadvantaged backgrounds who are likely to experience less and lower-quality language input (e.g., Hirsh-Pasek et al., 2015a) may benefit from video chat reading experiences, perhaps facilitated by programs that pair children with adult volunteers or through educators organizing virtual reading sessions. Providing children from low-SES backgrounds with opportunities to read with a caring adult virtually is extremely relevant, as many children are currently home without the resources to continue learning as they did in schools. Although the gap has narrowed somewhat in recent years, families experiencing poverty are still less likely to have access to books and engage in fewer bouts of shared book reading (Bassok et al., 2016). Caregivers of children in poverty often have less time to spend reading to their children (Neuman and Celano, 2001), with a 2017 report finding that 53% of low-SES children read or are read to every day compared to 68% of children from high-SES families (Rideout, 2017). Low-SES caregivers are also less likely to read in a style that is related to positive child language outcomes (van Kleeck, 1998; Berkule et al., 2007; Bornstein and Putnick, 2012). Thus, especially while schools and daycares are closed, preschoolers from low-income homes may be in an ideal position to profit from engaging in video chat storybook reading with a volunteer reader or a teacher. Indeed, the current findings suggest that 4-year-olds may benefit from reading over video chat with volunteers trained to use dialogic reading practices. Although programs like Jumpstart have demonstrated effectiveness by pairing children with adult mentors to work on reading through in-person experiences (Jumpstart, 2018), remote reading over video chat may have some additional advantages. It could alleviate barriers to volunteering by allowing participants to engage with children without the inconvenience of spending time in travel to a childcare site or to children’s homes (e.g., Sundeen and Raskoff, 2000). In the current moment, video chat reading would circumvent concerns about the spread of COVID-19.

Notably, based on the current findings, these advantages of video chat may also apply equally to prerecorded storybook reading. Indeed, it is promising that 4-year-olds in the current study learned equally well from a prerecorded video, which could be easily scaled and does not require additional time from an adult for each reading session. However, although more research is needed, we expect that over multiple sessions, children may benefit more from video chat reading due to the responsive feedback and the presence of a caring adult who can learn about the child’s skills and interests and tailor the reading experience.

### Limitations and Future Directions

Although the current findings are promising, some limitations must be considered. First, our sample was largely homogeneous. Given that children from disadvantaged backgrounds are likely to have lower cognitive and academic skills than their wealthier group counterparts (e.g., Morgan et al., 2011), children from more disadvantaged backgrounds might need more support than the children in the current sample. Like the younger children in prior studies (e.g., Parish-Morris et al., 2013; Kirkorian et al., 2016), children who come into the reading experience with lower levels of cognitive skills or less familiarity with book reading and dialogic reading practices may benefit from the socially contingent interactions in the Live and Video chat reading or from the social cues present in the Live condition specifically, and not perform as well in the Prerecorded condition. An additional limitation is that the current study was conducted in controlled settings, within quiet rooms containing minimal distractions. While children appeared to comprehend the prerecorded video without having an adult keep them on task or redirect their attention, a more naturalistic setting, such as the home or a classroom, may require a responsive adult to keep children engaged with the reading activity.

Future research should also assess which elements of the prerecorded video are essential to maintain high levels of comprehension. For example, research should disentangle the importance of a prerecorded video including a reader who directly addresses questions toward the camera, who uses dialogic reading prompts, and who pauses for potential responses from viewers. Additionally, it would be helpful to investigate if the style of video we used (i.e., minimal flashy or potentially distracting multimedia features) is the only type that promotes learning. Previous research suggests that these features can be either distracting or supportive depending on children’s age (Parish-Morris et al., 2013). The effect of these features on children’s learning also may depend on the nature of the feature (i.e., multimedia vs. interactive elements). One meta-analysis of e-books suggests that preschoolers and kindergarteners benefit from multimedia features, such as animations and sound effects, triggered by the story narration, rather than children’s touch (Takacs et al., 2015). However, the meta-analysis suggests that children may be distracted by interactive hotspots and games in the book. Further research in a prerecorded book reading context is warranted. Research should also test whether the prerecorded video maintains children’s attention and contingent book reading formats. Over multiple reading sessions, children may require additional prompts to stay focused on the story. In contingent reading sessions, the reader can use strategies, such as asking the child a question, to re-engage children’s attention if it falters. As the novelty of reading over a tablet fades over multiple sessions, children may become less attentive to the prerecorded video over time. Identifying the essential components of shared book reading is a crucial next step for promoting literacy and language learning in an increasingly digital age. Further research will help elucidate what specific components of book reading activities are essential for learning.

## Conclusion

Despite these limitations, the findings of the current study provide insight into 4-year-olds’ flexibility in understanding stories from different types of book reading activities. Specifically, in addition to comprehending books from live reading experiences, 4-year-olds reading with an adult over video chat, and even watching a video of an adult reading to them, also prospered. During the COVID-19 school and daycare closures, children may be exposed to more screen time than ever before. The current study provides some positive evidence that watching a video of book reading or reading over video chat can be an educational, engaging activity for children during the pandemic and beyond. When used thoughtfully, media and technology can facilitate the type of traditional shared reading that is the gold standard educational activity for young children.

## Data Availability Statement

The raw data supporting the conclusions of this article will be made available by the authors, without undue reservation.

## Ethics Statement

The studies involving human participants were reviewed and approved by University of Delaware Institutional Review Board and Purdue University Research Protection Program and Institutional Review Board. Written informed consent to participate in this study was provided by the participants’ legal guardian/next of kin.

## Author Contributions

YK, RD, DN, and RG conceptualized the idea for the project. YK, RD, and HP developed the study methodology and materials. CG, YK, HP, and research staff collected the data. CG, YK, HP, and research assistants coded the data. CG conducted statistical analyses and wrote the first draft. RD and YK provided critical feedback and edits. All authors contributed to the article and approved the submitted version.

### Conflict of Interest

The authors declare that the research was conducted in the absence of any commercial or financial relationships that could be construed as a potential conflict of interest.
